# Supplementary material for: From sequence to enzyme mechanism using multi-label machine learning
Source: BMC Bioinformatics. 2014 May 19;15:150. doi: 10.1186/1471-2105-15-150 (PMC4229970; doi:10.1186/1471-2105-15-150)
Supplement: Additional file 2 — Java code of ml2db. Additional file ml2db_code.tar.gz contains the Java source code to run the multi-label machine learning experiments and save the results to database. The code’s Javadoc is included. [file 1471-2105-15-150-S2.zip › additional file 2/ml2db/ml2db/doc/mulan/classifier/transformation/EnsembleOfClassifierChains.html]

EnsembleOfClassifierChains


JavaScript is disabled on your browser.


- Overview
- Package
- Class
- Use
- Tree
- Deprecated
- Index
- Help

- Prev Class
- Next Class

- Frames
- No Frames

- All Classes

- Summary:
- Nested |
- Field |
- Constr |
- Method

- Detail:
- Field |
- Constr |
- Method


mulan.classifier.transformation

## Class EnsembleOfClassifierChains

- java.lang.Object
- - mulan.classifier.MultiLabelLearnerBase
  - - mulan.classifier.transformation.TransformationBasedMultiLabelLearner
    - - mulan.classifier.transformation.EnsembleOfClassifierChains

- All Implemented Interfaces:
  :   java.io.Serializable, MultiLabelLearner, weka.core.TechnicalInformationHandler

  ---

    

  ```
  public class EnsembleOfClassifierChains
  extends TransformationBasedMultiLabelLearner
  ```

  Class implementing the Ensemble of Classifier Chains(ECC) algorithm. For more information, see

  Version:
  :   2012.02.27

  Author:
  :   Eleftherios Spyromitros-Xioufis ( espyromi@csd.auth.gr ), Konstantinos Sechidis (sechidis@csd.auth.gr), Grigorios Tsoumakas (greg@csd.auth.gr)

  See Also:
  :   Serialized Form

- - ### Constructor Summary

    Constructors

    Constructor and Description

    `EnsembleOfClassifierChains()`

    Default constructor

    `EnsembleOfClassifierChains(weka.classifiers.Classifier classifier,
    int aNumOfModels,
    boolean doUseConfidences,
    boolean doUseSamplingWithReplacement)`

    Creates a new object
  - ### Method Summary

    Methods

    | Modifier and Type | Method and Description |
    | --- | --- |
    | `int` | `getBagSizePercent()` Returns the size of each bag sample, as a percentage of the training size |
    | `double` | `getSamplingPercentage()` Returns the sampling percentage |
    | `weka.core.TechnicalInformation` | `getTechnicalInformation()` Returns an instance of a TechnicalInformation object, containing detailed information about the technical background of this class, e.g., paper reference or book this class is based on. |
    | `java.lang.String` | `globalInfo()` Returns a string describing classifier. |
    | `void` | `setBagSizePercent(int bagSizePercent)` Sets the size of each bag sample, as a percentage of the training size |
    | `void` | `setSamplingPercentage(double samplingPercentage)` Sets the sampling percentage |

    - ### Methods inherited from class mulan.classifier.transformation.TransformationBasedMultiLabelLearner

      `getBaseClassifier`
    - ### Methods inherited from class mulan.classifier.MultiLabelLearnerBase

      `build, getDebug, isUpdatable, makeCopy, makePrediction, setDebug`
    - ### Methods inherited from class java.lang.Object

      `equals, getClass, hashCode, notify, notifyAll, toString, wait, wait, wait`

- - ### Constructor Detail


    - #### EnsembleOfClassifierChains

      ```
      public EnsembleOfClassifierChains()
      ```

      Default constructor


    - #### EnsembleOfClassifierChains

      ```
      public EnsembleOfClassifierChains(weka.classifiers.Classifier classifier,
                                int aNumOfModels,
                                boolean doUseConfidences,
                                boolean doUseSamplingWithReplacement)
      ```

      Creates a new object

      Parameters:
      :   `classifier` - the base classifier for each ClassifierChain model
      :   `aNumOfModels` - the number of models
      :   `doUseConfidences` -
      :   `doUseSamplingWithReplacement` -
  - ### Method Detail


    - #### getBagSizePercent

      ```
      public int getBagSizePercent()
      ```

      Returns the size of each bag sample, as a percentage of the training size

      Returns:
      :   the size of each bag sample, as a percentage of the training size


    - #### setBagSizePercent

      ```
      public void setBagSizePercent(int bagSizePercent)
      ```

      Sets the size of each bag sample, as a percentage of the training size

      Parameters:
      :   `bagSizePercent` - the size of each bag sample, as a percentage of
          the training size


    - #### getSamplingPercentage

      ```
      public double getSamplingPercentage()
      ```

      Returns the sampling percentage

      Returns:
      :   the sampling percentage


    - #### setSamplingPercentage

      ```
      public void setSamplingPercentage(double samplingPercentage)
      ```

      Sets the sampling percentage

      Parameters:
      :   `samplingPercentage` - the sampling percentage


    - #### globalInfo

      ```
      public java.lang.String globalInfo()
      ```

      Returns a string describing classifier.

      **Overrides:**
      :   `globalInfo` in class `TransformationBasedMultiLabelLearner`

      Returns:
      :   a description suitable for displaying in the
          explorer/experimenter gui


    - #### getTechnicalInformation

      ```
      public weka.core.TechnicalInformation getTechnicalInformation()
      ```

      **Description copied from class: `TransformationBasedMultiLabelLearner`**

      Returns an instance of a TechnicalInformation object, containing detailed
      information about the technical background of this class, e.g., paper
      reference or book this class is based on.

      **Specified by:**
      :   `getTechnicalInformation` in interface `weka.core.TechnicalInformationHandler`

      **Overrides:**
      :   `getTechnicalInformation` in class `TransformationBasedMultiLabelLearner`

      Returns:
      :   the technical information about this class


- Overview
- Package
- Class
- Use
- Tree
- Deprecated
- Index
- Help

- Prev Class
- Next Class

- Frames
- No Frames

- All Classes

- Summary:
- Nested |
- Field |
- Constr |
- Method

- Detail:
- Field |
- Constr |
- Method
